# Factors associated with levothyroxine withdrawal and development of a prediction model in primary hypothyroidism

**DOI:** 10.3389/fendo.2026.1852620

**Published:** 2026-07-21

**Authors:** Wanli Zheng, Yibei Tang, Xuanyu Chen, Wang Ye, Jingyao Yu, Yihan Sun, Bin Zhang, Guangli Wu, Jipeng Zheng, Yuqing Wang, Jianchun Cui, Li Lu, Xingai Ju

**Affiliations:** 1 Department of General Medicine, People's Hospital of China Medical University (People's Hospital of Liaoning Province), Shenyang, China; 2 Jinzhou Medical University, Jinzhou, China; 3 China Medical University, Shenyang, China; 4 Department of Emergency, People's Hospital of China Medical University (People's Hospital of Liaoning Province), Shenyang, China; 5 Dalian Medical University, Dalian, China; 6 Department of Cardiology, People's Hospital of China Medical University (People's Hospital of Liaoning Province), Shenyang, China; 7 Department of Thyroid and Breast Surgery, People's Hospital of China Medical University (People's Hospital of Liaoning Province), Shenyang, China; 8 Department of Endocrinology, People's Hospital of China Medical University (People's Hospital of Liaoning Province), Shenyang, China

**Keywords:** levothyroxine, nomogram, prediction model, primary hypothyroidism, withdrawal

## Abstract

**Objective:**

To identify factors associated with successful levothyroxine (L-T4) withdrawal in patients with primary hypothyroidism and to develop a clinical prediction model.

**Methods:**

We retrospectively included patients with primary hypothyroidism who received L-T4 treatment at our hospital between January 2016 and October 2024 and had a documented withdrawal attempt with follow-up records. Baseline patient data were collected, including sociodemographic characteristics (age, sex, body mass index [BMI], family history of thyroid disease), medication-related indices (L-T4 dosage and treatment duration), clinical classification (subclinical vs. overt hypothyroidism), and laboratory and ultrasound imaging parameters (TSH, FT3, FT4, TPOAb, TgAb, heterogeneous echotexture, and hypoechogenicity). Successful withdrawal was defined as maintenance of euthyroidism for 12 months after stopping L-T4; otherwise, withdrawal was considered unsuccessful. Candidate predictors were screened using univariate logistic regression and entered into multivariable logistic regression to identify independent predictors and construct a nomogram. Discriminative ability was assessed using the area under the receiver operating characteristic curve (AUC). Calibration and internal validation were evaluated using the Hosmer-Lemeshow test and bootstrap resampling (1,000 iterations).

**Results:**

A total of 110 patients were included, of whom 39 (35.5%) achieved successful withdrawal and 71 (64.5%) did not. In multivariable analysis, younger age (OR = 0.901, 95% CI: 0.852–0.953, P < 0.001) and subclinical hypothyroidism (OR = 4.879, 95% CI: 1.343–17.723, P = 0.016) were associated with a higher likelihood of successful withdrawal. In contrast, TPOAb positivity (OR = 0.150, 95% CI: 0.040–0.556, P = 0.005) and heterogeneous thyroid echotexture on ultrasound (OR = 0.155, 95% CI: 0.032–0.751, P = 0.021) were associated with a lower likelihood of success. The model showed excellent discrimination (AUC = 0.899, 95% CI: 0.840–0.957, P < 0.001). At the optimal cutoff value of 0.292, sensitivity was 87.2% and specificity was 80.3%. Model fit was good (Hosmer-Lemeshow χ² = 5.448, P = 0.709), and bootstrap validation demonstrated good calibration (mean absolute error = 0.029).

**Conclusion:**

Younger age, subclinical hypothyroidism, negative TPOAb, and homogeneous thyroid echotexture were associated with a higher likelihood of successful L-T4 withdrawal. The nomogram model may facilitate pre-withdrawal risk stratification and individualized follow-up planning, supporting quantitative decision-making in clinical practice.

## 1 Background

Primary hypothyroidism is a common endocrine disorder resulting from structural or functional impairment of the thyroid gland, leading to insufficient thyroid hormone (TH) production and a generalized slowing of metabolism with multisystem manifestations (1). Typical symptoms include fatigue, cold intolerance, edema, weight gain, depressed mood, and cognitive complaints such as memory impairment (2). Levothyroxine (L-T4) is the standard replacement therapy; it is peripherally converted to biologically active hormone triiodothyronine (T3) and effectively corrects symptomatic and biochemical abnormalities related to TH deficiency in most patients (3).

Long-term L-T4 therapy requires ongoing dose titration, periodic monitoring, and sustained adherence. In routine practice, however, patients and clinicians may face difficulties related to complex medication management, quality of life concerns, and variability in how treatment benefit is assessed (4–7). In older adults, physiological TH requirements often decline with age, consequently, fixed or unchanged maintenance doses may lead to low or suppressed serum TSH, increasing the risk of iatrogenic hyperthyroidism (8). Over-replacement or inappropriate L-T4 has been associated with adverse outcomes, including atrial fibrillation and cardiac ischemic symptoms such as angina, as well as accelerated bone loss and an increased risk of fracture (9–11). Notably, a substantial proportion of patients initiated on L-T4 have baseline TSH levels within the normal range, reflecting a pattern of potentially inappropriate prescribing (12). Importantly, such populations typically show higher withdrawal success rates, as their underlying thyroid function may have been near-normal or only transiently impaired.

Current guidelines suggest that the benefit of thyroid hormone therapy for subclinical hypothyroidism is limited to adults under 65 years with TSH levels between 4.5 and 10 mIU/L (13). For older adults (≥65 years) or those with TSH levels >10 mIU/L, the evidence is less conclusive, and treatment decisions should be individualized. Accordingly, systematic evaluation of whether long-term replacement therapy is necessary – and when a supervised withdrawal trial can be considered – has important clinical implications. Nevertheless, available studies indicate that approximately 20–50% of patients can maintain stable thyroid function after stopping L-T4, with higher rates reported among those with subclinical hypothyroidism (8, 14–21).

In recent years, research on whether patients with primary hypothyroidism can safely discontinue L-T4 has gradually increased. International evidence suggests that thyroid function may normalize spontaneously in some patients – particularly those with transient thyroiditis or subclinical hypothyroidism (5, 22–25). A recent randomized controlled trial further supported the feasibility and safety of L-T4 withdrawal in adults with subclinical hypothyroidism (14). Accurately identifying patients with a high likelihood of maintaining euthyroidism after withdrawal could reduce unnecessary long-term drug exposure, mitigate treatment-related adverse risks, and lessen both financial costs and follow-up burden. These observations suggest that a subset of patients may have reversible hypothyroidism or may warrant reassessment of treatment indications, highlighting the need for structured withdrawal evaluation strategies and risk stratification tools (26, 27). At present, identifying candidates for L-T4 withdrawal remains challenging, and quantitative decision aids are limited. Therefore, we aimed to systematically evaluate factors associated with successful L-T4 withdrawal in patients with hypothyroidism, and to develop a clinically applicable prediction model supporting individualized risk assessment and treatment optimization.

## 2 Materials and methods

### 2.1 Study design and participants

This was a single-center retrospective cohort study conducted at Liaoning Provincial People’s Hospital. We included adult patients (≥18 years) diagnosed with primary hypothyroidism who received levothyroxine (L-T4) treatment between January 2016 and October 2024. All enrolled patients had a documented attempt to withdraw L-T4 under physician guidance and were followed for 12 months after drug withdrawal. Based on the 12-month outcome, they were divided into the successful withdrawal group and the unsuccessful withdrawal group for comparative analysis.

Diagnosis of primary hypothyroidism was confirmed by reviewing electronic medical records and discharge summaries, defined according to the 2017 Chinese Guidelines for the Diagnosis and Treatment of Adult Hypothyroidism (28), requiring biochemical evidence of TSH elevation with or without FT4 reduction, with no identifiable pituitary or hypothalamic pathology.

Patients were excluded if they had: (1) secondary or central hypothyroidism; (2) prior thyroid surgery or radioiodine therapy; (3) severe systemic conditions potentially affecting thyroid function (e.g., liver or renal failure, severe infection, malignancy); (4) pregnancy or within 12 months postpartum; (5) a previous diagnosis of other thyroid diseases, especially Graves’ disease or subacute thyroiditis; (6) use of medications known to interfere with the hypothalamic–pituitary–thyroid axis (e.g., glucocorticoids, amiodarone, lithium, oral contraceptives, metformin) when their effects could not be excluded or reliably accounted for; or (7) missing key laboratory or follow-up data.No minimum treatment duration or L-T4 maintenance dosage was stipulated as an inclusion criterion; all patients who met the diagnostic and follow-up requirements and had a documented physician-guided withdrawal attempt were eligible regardless of the duration or dose of prior therapy.

The study protocol was approved by the Ethics Committee of Liaoning Provincial People’s Hospital (Approval No.: (2025) K007).

### 2.2 Data collection

Clinical data were extracted from the electronic medical record system. Variables collected included gender, age, body mass index (BMI), family history of thyroid diseases, hypothyroidism classification (subclinical/clinical), thyroid function parameters (free triiodothyronine [FT3], free thyroxine [FT4], thyroid-stimulating hormone [TSH]) assessed before withdrawal and at the time of withdrawal, thyroid autoantibodies (thyroid peroxidase antibody [TPOAb], thyroglobulin antibody [TgAb]), thyroid ultrasound features (heterogeneous echotexture and hypoechogenicity), L-T4 dose, and duration of L-T4 therapy.

### 2.3 Diagnostic criteria

The diagnostic criteria and classification of primary hypothyroidism in this study were based on the Guidelines for the Diagnosis and Treatment of Adult Hypothyroidism (2017). The definitions were as follows:

1. Subclinical hypothyroidism (SCH): Elevated serum TSH levels with FT4 levels within the normal reference range.
2. Overt hypothyroidism (OH): Elevated serum TSH levels accompanied by decreased FT4 levels.

All laboratory tests were conducted at our institutional laboratory. Serum FT3, FT4, TSH, TPOAb, and TgAb levels were measured using direct chemiluminescence immunoassay on a Siemens Atellica IM 1600 automated analyzer with manufacturer-provided reagents. The reference ranges (established by our hospital laboratory) were as follows: FT3, 2.30–4.20 pg/mL; FT4, 0.89–1.76 ng/dL; TSH, 0.55–4.78 mIU/L; TPOAb, 0–60 IU/mL; TgAb, 0–4.5 IU/mL.

### 2.4 Withdrawal protocol

As this was a retrospective cohort study, L-T4 withdrawal was not governed by a pre-specified, uniform protocol. The following describes withdrawal practices as ascertained from systematic retrospective chart review.

Initiation of withdrawal. In all documented cases, withdrawal was confirmed to have been initiated under physician guidance rather than by patients independently. This was verified by cross-referencing outpatient visit records, prescribing histories, and documented clinical decision notes.

Tapering approach. Dose adjustment was performed every 3 months. At each step, the current daily dose of L-T4 was reduced by one-quarter of a tablet (equivalent to 12.5 μg, assuming a standard 50−μg tablet strength) until the dose reached one-quarter tablet (12.5 μg/day). This minimal dose was then maintained for an additional 3 months, after which L-T4 was completely discontinued.

Pre-withdrawal thyroid function status. Consistent with standard clinical practice at our institution, patients were generally considered candidates for withdrawal only after serum TSH had been maintained within the institutional reference range (0.55–4.78 mIU/L) on a stable L-T4 dose for a minimum period of approximately 6 months. However, owing to the retrospective design, the exact duration of pre-withdrawal euthyroidism was not prospectively and uniformly documented across all patients. Variability in the pre-withdrawal stabilization period may have contributed to heterogeneity in withdrawal eligibility criteria across treating physicians, introducing a degree of implementation bias that cannot be fully controlled for in the present analysis.

Post-withdrawal follow-up. Post-withdrawal thyroid function monitoring was conducted according to a standardized schedule. Serum TSH, FT3, and FT4 were assessed at fixed time points of 3, 6, and 12 months after drug discontinuation, primarily through scheduled outpatient visits supplemented by telephone contact where necessary.

### 2.5 Outcome definition and follow-up

The primary outcome was successful L-T4 withdrawal, defined by maintenance of euthyroid thyroid function for 12 months after stopping L-T4. Patients were classified as follows:

1. Successful withdrawal group: TSH, FT3, and FT4 remained within the laboratory reference ranges throughout 12 consecutive months of follow-up, and L-T4 was not restarted. Additionally, pharmacy prescription records were cross-referenced to confirm the absence of any thyroid hormone reissue during the entire follow-up period.
2. Unsuccessful withdrawal group: Within 12 months after withdrawal, thyroid function became abnormal (TSH elevation and/or FT3 and FT4 reduction, with or without recurrent hypothyroid symptoms, and/or L-T4 therapy was restarted based on endocrinologist assessment).

Follow-up procedures and time points are described in Section 1.4. Patients with loss to follow-up or missing critical data were excluded per the exclusion criteria to ensure the completeness and integrity of outcome data.

### 2.6 Statistical analysis

Statistical analyses were performed using SPSS (version 27.0) and R (version 4.5.0). Continuous variables were presented as mean ± standard deviation (SD) for normally distributed data, and compared using the Student’s t-test; non-normally distributed variables were presented as median [interquartile range (IQR)], and compared using the Mann-Whitney U test. Categorical variables were presented as counts (percentage) and compared using the χ² test.

Univariable logistic regression was used to screen candidate predictors of successful withdrawal. Using successful L-T4 withdrawal as the outcome (unsuccessful=0, successful=1), we performed univariate logistic regression analyses including hypothyroidism classification, age, gender, BMI, family history, FT3, FT4, TSH, TgAb status, TPOAb status, thyroid ultrasound features (heterogeneous echotexture and hypoechogenicity), L-T4 dose, and treatment duration. Variables with statistical significance in univariable analyses were entered into a multivariate logistic regression model to identify independent predictors. A nomogram was developed based on the final multivariate model. Model discrimination was assessed using the receiver operating characteristic curve (AUC). Calibration and goodness-of-fit were evaluated using the Hosmer-Lemeshow test. Internal validation was performed using Bootstrap resampling (1,000 iterations) to generate a calibration curve and estimate the mean absolute error (MAE). Effect sizes are reported as odds ratios (ORs) with 95% confidence intervals (CIs). A two-sided P value < 0.05 was considered statistically significant.

Based on the final multivariate logistic regression model, independent predictors were incorporated to estimate the probability of successful levothyroxine (L-T4) withdrawal in patients with primary hypothyroidism. The coefficients of the final prediction model are presented in **Supplementary Table 2**.

Variable definitions were as follows:

- X_1_: Subclinical hypothyroidism (Yes=1, No=0 [overt hypothyroidism as reference])
- X_2_: TPOAb positivity (Positive=1, Negative=0)
- X_3_: Heterogeneous thyroid echotexture on ultrasound (Yes=1, No=0)
- Age: Continuous (years)

## 3 Results

### 3.1 Baseline characteristics

A total of 110 patients with primary hypothyroidism were included, comprising 83 women (75.5%) and 27 men (24.5%). Based on thyroid function status during the 12-month follow-up after levothyroxine (L-T4) withdrawal, 39 patients (35.5%) were classified as the successful withdrawal group and 71 patients (64.5%) as the unsuccessful withdrawal group.

Compared with the unsuccessful withdrawal group, the successful withdrawal group had a higher proportion of subclinical hypothyroidism (66.7% vs. 29.6%, P < 0.001) and was younger (54.23 ± 12.71 vs. 66.15 ± 10.97 years, P < 0.001). At initial diagnosis, prior to L-T4 initiation, the successful withdrawal group had a lower baseline TSH level (median [IQR], 5.89 [4.81, 9.21] vs. 12.67 [6.35, 23.45] mIU/L, P < 0.001), and lower positivity rates for TgAb (61.5% vs. 80.3%, P = 0.033) and TPOAb (43.6% vs. 71.8%, P = 0.004). On ultrasound, heterogeneous echotexture (48.7% vs. 88.7%, P < 0.001) and hypoechogenicity (33.3% vs. 69.0%, P < 0.001) were less frequent in the successful withdrawal group. No significant between-group differences were observed in gender, BMI, family history, FT3, FT4, L-T4 dose, or treatment duration (all P > 0.05) (**Table 1**).

**Table 1:** Baseline characteristics of patients with primary hypothyroidism according to L-T4 withdrawal outcome.

Clinical data	Unsuccessful withdrawal group (n=71)	Successful withdrawal group (n=39)	Statistic (Z/t/χ^2^)	P value
Classification [n(%)]			14.151	< 0.001
Subclinical hypothyroidism	21 (29.6)	26 (66.7)		
Overt hypothyroidism	50(70.4)	13 (33.3)		
Age (years)	66.15 ± 10.97	54.23 ± 12.71	5.151	< 0.001
Gender [n(%)]			1.420	0.233
Male	20 (28.2)	7 (17.9)		
Female	51 (71.8)	32 (82.1)		
BMI(kg/m^2^)	23.94 ± 3.27	24.12 ± 3.06	0.279	0.781
Family History [n(%)]			0.252	0.615
No	64 (90.1)	37 (94.9)		
Yes	7 (9.9)	2 (5.1)		
FT3(pg/mL)	2.65 ± 0.55	2.54 ± 0.74	0.842	0.403
FT4(ng/dL)	0.92 ± 0.30	0.92 ± 0.31	0.125	0.901
TSH(mIU/L)	12.67 (6.35,23.45)	5.89 (4.81,9.21)	-4.174	< 0.001
TgAb [n(%)]			4.555	0.033
Negative	14 (19.7)	15 (38.5)		
Positive	57 (80.3)	24 (61.5)		
TPOAb [n(%)]			8.506	0.004
Negative	20 (28.2)	22 (56.4)		
Positive	51 (71.8)	17 (43.6)		
Heterogeneous echotexture [n(%)]			21.241	< 0.001
No	8 (11.3)	20 (51.3)		
Yes	63 (88.7)	19 (48.7)		
Hypoechogenicity [n(%)] No Yes	22 (31.0) 49 (69.0)	26 (66.7) 13 (33.3)	13.030	< 0.001
Treatment Dose (μg)	50.00 (25.00,75.00)	50.00 (25.00,50.00)	-1.747	0.081
Treatment Duration (months)	26.00 (18.00,48.00)	22.00 (16.00,36.00)	-1.329	0.184

Data are presented as mean ± SD for normally distributed variables, median (IQR) for non-normally distributed variables, and n(%) for categorical variables. FT3, free triiodothyronine; FT4, free thyroxine; TSH, thyroid-stimulating hormone; TPOAb, thyroid peroxidase antibody; TgAb, thyroglobulin antibody. Reference ranges: FT3 2.30–4.20 pg/mL, FT4 0.89–1.76 ng/dL, TSH 0.55–4.78 mIU/L.

Notably, baseline TSH in the unsuccessful withdrawal group showed a wide distribution, with a median of 12.67 mIU/L (IQR: 6.35–23.45), indicating that the majority of patients in this group had initial TSH levels exceeding 10 mIU/L at the time of diagnosis—a threshold generally associated with more pronounced thyroid dysfunction and lower likelihood of spontaneous functional recovery. The clinical implications of this cohort characteristic are further discussed in Section 3.5.

The cohort-wide low L-T4 maintenance dose (median: 50 μg/day) and short treatment duration (median: 22–26 months) indicate that many patients were recently initiated on low-dose therapy and had not yet reached full replacement—consistent with an early dose-titration phase or preserved residual thyroid function, even among those classified as overt hypothyroidism (for whom full replacement typically requires 75–125 μg/day). No minimum dose or treatment duration was specified in the inclusion criteria; these are therefore intrinsic cohort characteristics rather than a deliberate selection strategy. The generalizability implications are discussed in Section 3.7.

### 3.2 Univariable logistic regression analysis

Hypothyroidism classification, age, TSH, TgAb, TPOAb, heterogeneous echotexture, and hypoechogenicity were significantly associated with successful withdrawal (P < 0.05)(**Table 2**).

**Table 2:** Univariate logistic regression analysis for successful L-T4 withdrawal in primary hypothyroidism.

Factor	B	SE	Wald χ^2^	P	OR (95% CI)
Hypothyroidism Classification (subclinical vs overt)	1.561	0.428	13.309	< 0.001	4.762 (2.059–11.013)
Age	-0.089	0.022	16.752	< 0.001	0.915 (0.877–0.955)
Gender	0.584	0.494	1.398	0.237	1.793 (0.681–4.718)
BMI	0.018	0.063	0.079	0.779	1.018 (0.9–1.151)
Family History	-0.705	0.828	0.725	0.395	0.494 (0.098–2.504)
FT3	-0.295	0.322	0.842	0.359	0.744 (0.396–1.398)
FT4	0.083	0.660	0.016	0.900	1.087 (0.298–3.958)
TSH	-0.046	0.019	5.649	0.017	0.955 (0.919–0.992)
TgAb positivity	-0.934	0.444	4.421	0.035	0.393 (0.165–0.939)
TPOAb positivity	-1.194	0.417	8.198	0.004	0.303 (0.134–0.686)
Heterogeneous echotexture (yes vs no)	-2.115	0.493	18.370	< 0.001	0.121 (0.046–0.317)
Hypoechogenicity (yes vs no)	-1.494	0.426	12.314	< 0.001	0.224 (0.097–0.517)
L-T4 Dose	-0.015	0.008	3.452	0.063	0.985 (0.969–1.001)
Treatment Duration	-0.007	0.008	0.770	0.380	0.993 (0.977–1.009)

B, regression coefficient; SE, standard error; Wald χ², Wald chi-square value; OR, odds ratio; 95%CI, 95% confidence interval.

Specifically, subclinical hypothyroidism was associated with a higher likelihood of successful withdrawal (OR = 4.762, 95%CI: 2.059–11.013, P < 0.001). Increasing age was associated with lower odds of success (OR = 0.915 per year, 95%CI: 0.877–0.955, P< 0.001). TPOAb positivity (OR = 0.303, 95%CI: 0.134–0.686, P = 0.004) and heterogeneous echotexture on ultrasound (OR = 0.121, 95%CI: 0.046–0.317, P < 0.001) were associated with reduced odds of successful withdrawal.

### 3.3 Multivariable logistic regression analysis

Variables with statistically significance in the univariate analysis were entered into a multivariate logistic regression model (Variables were coded as shown in **Supplementary Table 1**). In the multivariable model, subclinical hypothyroidism (vs. overt hypothyroidism) was independently associated with a higher likelihood of successful L-T4 withdrawal (OR = 4.879, 95%CI: 1.343–17.723, P = 0.016). Increasing age was associated with reduced odds of success (OR = 0.901, 95%CI: 0.852–0.953, P < 0.001). TPOAb positivity (OR = 0.150, 95%CI: 0.040–0.556, P = 0.005) and heterogeneous thyroid echotexture on ultrasound (OR = 0.155, 95%CI: 0.032–0.751, P = 0.021) were independently associated with lower odds of successful withdrawal (**Supplementary Table 3**).

### 3.4 Development of the Prediction Model and Nomogram

The model was specified as:


$$\text{Logit}\left(\text{P}\right)=7.449\;+\;1.853\times {\text{X}}_{1}-0.103\times \text{Age}-1.748\times {\text{X}}_{2}-2.213\times {\text{X}}_{3}$$

where P represents the probability of successful L-T4 withdrawal and is calculated as:


$$\text{P}=1/\left(1+e^{\wedge }\left(-\text{Logit}\left(\text{P}\right)\right)\right)$$

**Figure 1:**
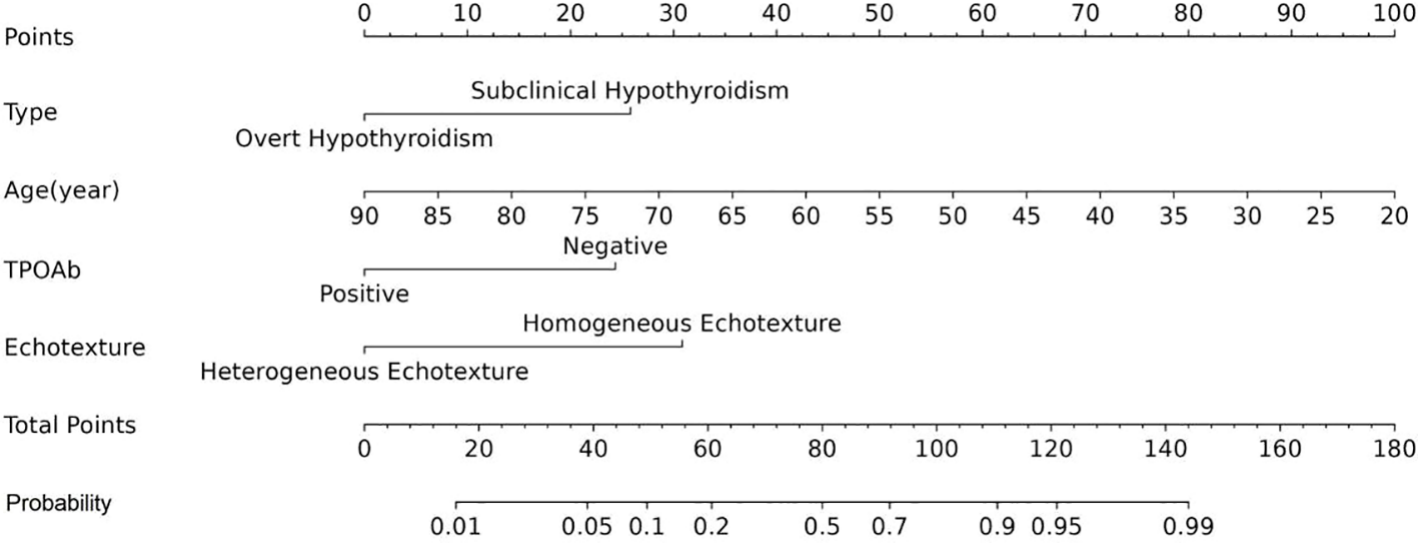
Nomogram for predicting the probability of successful L-T4 withdrawal in primary hypothyroidism.

A nomogram was constructed based on the regression model (**Figure 1**) to provide an individualized estimate of the probability of successful L-T4 withdrawal. The nomogram incorporates four predictors— age, hypothyroidism classification, TPOAb status, and thyroid ultrasound echotexture. Each predictor is assigned a point value; the sum of the individual points yields the Total Points, which corresponds to the estimated probability of successful withdrawal on the probability scale. The nomogram visually indicates that younger patients, those with subclinical hypothyroidism, negative TPOAb, and a homogeneous ultrasound echotexture are more likely to achieve successful L-T4 withdrawal.

### 3.5 Model performance

**Figure 2:**
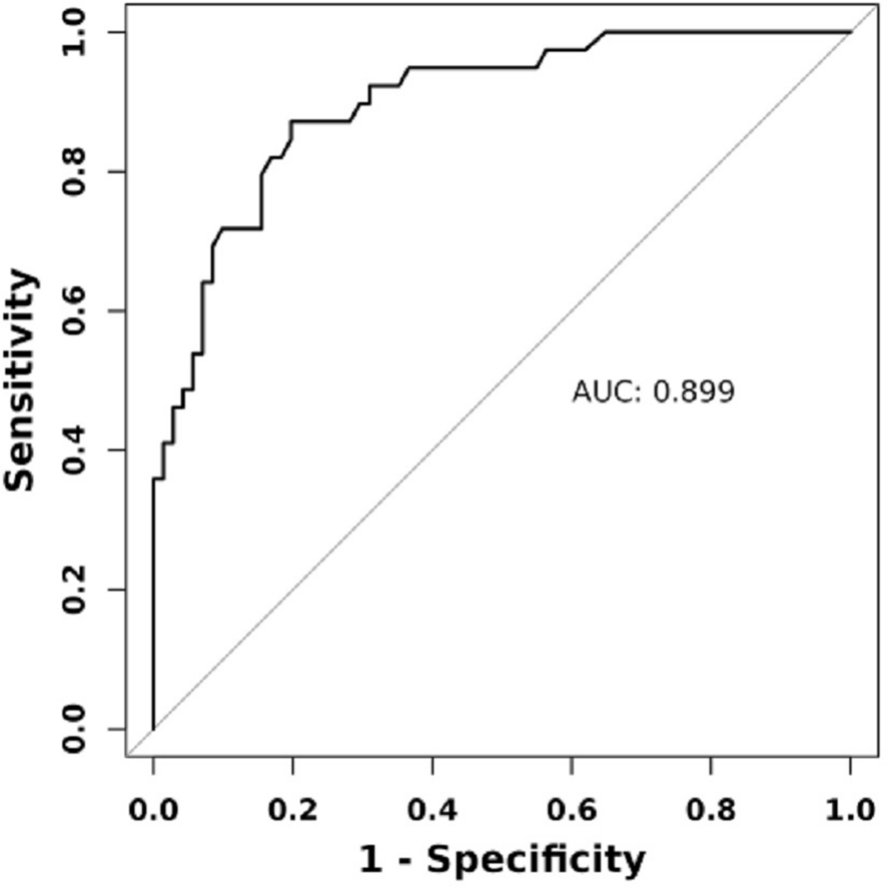
Receiver operating characteristic (ROC) curve for the prediction model. The ROC curve assesses the discriminative ability of the model to distinguish between patients with successful and unsuccessful L-T4 withdrawal. The area under the curve (AUC) is 0.899 (95% confidence interval [CI]: 0.840–0.957, P<0.001), indicating excellent discriminative performance. At the optimal cutoff value of 0.292, the model achieves a sensitivity of 87.2% and a specificity of 80.3%.

**Figure 3:**
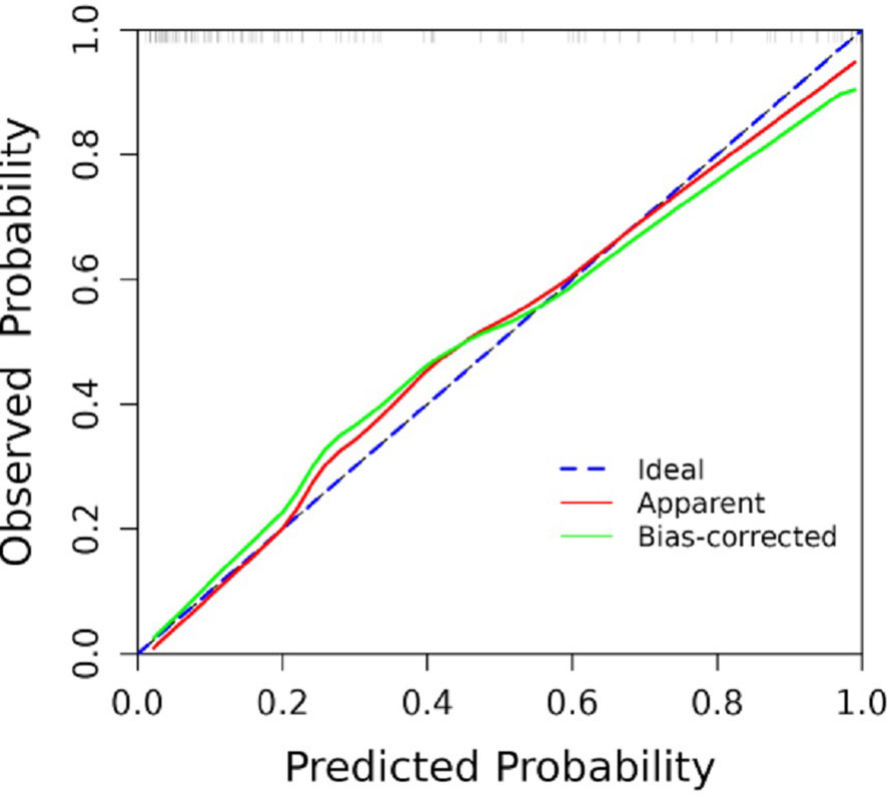
Calibration curve of the prediction model with bootstrap validation (1,000 resamples). The calibration curve evaluates the agreement between the predicted probability of successful L-T4 withdrawal and the observed actual outcome. The x-axis represents the predicted probability, and the y-axis represents the observed probability. Bootstrap resampling (1,000 iterations) was used for internal validation, with a mean absolute error (MAE) of 0.029, indicating good calibration of the model. The closer the curve is to the diagonal (45° line), the better the consistency between predicted and observed probabilities.

Model-predicted probabilities were calculated for each patient, and discrimination was evaluated using the receiver operating characteristic (ROC) (**Figure 2**). The model demonstrated excellent discrimination, with an AUC of 0.899 (95% CI: 0.840–0.957). Using an optimal cut-off probability of 0.292, the sensitivity was 87.2% and the specificity was 80.3%. Model calibration was assessed using the Hosmer-Lemeshow test, which indicated adequate fit (χ^2^ = 5.448, P = 0.709). Internal validation was performed using bootstrap resampling (1,000 iterations). The calibration curve showed good agreement between predicted and observed probabilities, with a mean absolute error (MAE) of 0.029 (**Figure 3**).

## 4 Discussion

Whether patients can safely withdraw levothyroxine (L-T4) after treatment is a key clinical decision in the management of primary hypothyroidism. Successful withdrawal not only suggests recovery or preservation of endogenous thyroid function but also reduces the financial, psychological, and monitoring burden associated with long-term replacement therapy (21). However, predictors of successful L-T4 withdrawal remain incompletely defined, and standardized assessment strategies are lacking. In this retrospective cohort of patients treated with L-T4 at our center between January 2016 and October 2024, 35.5% maintained euthyroidism for 12 months after withdrawal, consistent with the approximately one-third success rate reported in a previous systematic review (20). In multivariate analyses, hypothyroidism classification (subclinical hypothyroidism), age, TPOAb status, and thyroid ultrasound echotexture were independently associated with withdrawal outcome. The nomogram derived from these variables demonstrated excellent discrimination (AUC = 0.899) with good internal calibration, supporting its potential utility as a pragmatic tool for pre-withdrawal risk stratification.

### 4.1 Age: A key demographic predictor of successful withdrawal

Age emerged as an important demographic predictor of L-T4 withdrawal outcome in our cohort study. Although some studies have reported no significant association between age and withdrawal success (15, 18), we observed a consistent inverse relationship: patients in the successful withdrawal group were younger, and each additional year of age was associated with lower odds of success (OR = 0.903 per year, P < 0.001). This finding may reflect greater thyroid functional reserve and regenerative capacity in young individuals. With aging, physiological decline in thyroid reserve, cumulative autoimmune-mediated tissue injury, and reduced capacity for tissue repair may limit the potential for functional recovery (25). Moreover, older adults more frequently have chronic low-grade inflammation, comorbidities, and polypharmacy, which may further compromise the likelihood of maintaining euthyroidism after withdrawal (9).Notably, Kim et al. found that among children and adolescents with Hashimoto’s thyroiditis (HT), older age (≥12 years) was associated with a higher probability of successful withdrawal (21). This apparent discrepancy likely reflects fundamental differences in disease biology between pediatric and adult populations. In childhood-onset autoimmune thyroiditis, immune activity may be more dynamic, and younger children may experience more active or fluctuating immune-mediated thyroid injury, reducing the chance of spontaneous functional stabilization (22). In contrast, older children may enter a more stable phase of disease, increasing the likelihood of maintaining euthyroidism after treatment withdrawal.

Taken together, the influence of age on withdrawal success appears to be population-specific and should be interpreted in the context of age group, underlying etiology, and disease course when designing individualized withdrawal strategies.

It is worth noting that TSH levels rise physiologically with age, and standard reference ranges may not adequately account for this variation (29). For older adults, a mildly elevated TSH may fall within age-specific normal limits, suggesting that some patients classified as ‘unsuccessful withdrawal’ in this study—particularly those ≥65 years—may not have experienced clinically meaningful relapse. A sensitivity analysis applying age-adjusted TSH thresholds (e.g., a raised upper reference limit for adults ≥65 years) would be informative to assess whether withdrawal success rates differ when age-specific norms are applied. This represents an important analytical limitation and warrants further investigation in future studies.

### 4.2 Hypothyroidism classification: a marker of functional impairment and recoverability

In this study, subclinical hypothyroidism was strongly associated with successful L-T4 withdrawal (OR = 6.377). Subclinical hypothyroidism is characterized by modest TSH elevation with circulating thyroid hormone levels within the reference range, suggesting relatively preserved thyroid reserve and the absence of advanced, irreversible tissue damage (5). When transient triggers (e.g., acute illness or stress, pregnancy-related changes, or fluctuations in iodine intake) resolve, endogenous hypothalamic–pituitary–thyroid axis function may recover, enabling maintenance of euthyroidism without continued replacement therapy. Our findings align with prior evidence indicating that patients with subclinical hypothyroidism retain greater endogenous function and are more likely to remain stable following withdrawal (20). Prospective data also support the feasibility and safety of supervised L-T4 withdrawal in selected adults with subclinical hypothyroidism (16). These results underscore the importance of periodic re-evaluation of treatment indication in treated subclinical hypothyroidism and suggest that, when close follow-up is feasible, a risk-stratified withdrawal trial may reduce unnecessary long-term therapy and monitoring burden. Furthermore, a significant proportion of patients initiated on L-T4 in routine clinical practice have baseline TSH levels within the normal range at treatment initiation, suggesting diagnostic uncertainty or potentially inappropriate prescribing (12). Patients in this category are more likely to have reversible or transient hypothyroidism, which may partially explain the relatively high withdrawal success rate observed in our subclinical subgroup.

### 4.3 Ultrasonic features: independent prognostic value of structural abnormalities

Beyond its role in nodule assessment, thyroid ultrasound has increasing value in characterizing diffuse thyroid disease and informing long-term management decisions. Prior studies suggest that ultrasound evidence of diffuse thyroid disease (DT) is associated with persistent TSH elevation and poorer biochemical response in subclinical hypothyroidism (30). In our cohort study, heterogeneous echotexture was independently associated with markedly lower odds of successful withdrawal (OR = 0.109). This pattern likely reflects ongoing chronic inflammation, progressive fibrosis, and disruption of normal follicular architecture, which may constrain the capacity for endogenous functional recovery. Consistent with our findings, a recent prospective study identified diffuse heterogeneous echotexture as a key imaging feature indicating the need for long-term L-T4 therapy (15).

Although hypoechogenicity was not statistically significant in the multivariable model (P = 0.207), its OR value was still lower than 1 (OR = 0.453), suggesting a potential association that may become evident in larger cohorts. Ultrasound structural changes are closely linked to autoimmune activity; abnormal echotexture is more common in TPOAb-positive autoimmune thyroid disease (30). Importantly, imaging abnormalities may precede overt changes in circulating antibody levels, making ultrasound a practical and repeatable tool for longitudinal assessment.

### 4.4 TPOAb status: serologic marker of autoimmune activity

TPOAb positivity was independently associated with a lower likelihood of successful withdrawal (OR = 0.174), suggesting that patients with greater autoimmune activity are more likely to experience biochemical relapse after stopping L-T4. This observation is consistent with prior reports that higher autoantibody levels are associated with withdrawal failure (19). As a routinely available and reproducible biomarker, TPOAb provides clinically useful information on autoimmune-mediated thyroid injury and can be incorporated into risk assessment when considering a withdrawal trial.

### 4.5 TSH and treatment-related variables

Several studies have emphasized the role of TSH in predicting withdrawal success. Jung et al. found that adults who successfully discontinued L-T4 had a lower baseline TSH level at treatment initiation than those requiring ongoing therapy (18). Rosario et al. further suggested that an initial TSH<8 mIU/L at diagnosis was associated with long-term withdrawal success (16). Together, these support the concept that lower TSH is linked to a higher likelihood of successful withdrawal. In our univariable analyses, baseline TSH at initial diagnosis was associated with outcome; however, after adjustment for age, hypothyroidism classification, and ultrasound features, TSH was no longer an independent predictor. A single TSH measurement is susceptible to short-term influences such as medication compliance, timing of blood sampling, acute stress, and infection. Consistent with this, Livadas et al. found that ultrasound-based structural indicators may outperform TSH as predictors in withdrawal models (15). Clinically, these findings argue against over-reliance on a single TSH value when making withdrawal decisions. Future studies should evaluate whether incorporating longitudinal TSH trajectories, rather than a single time-point measure, improves predictive accuracy.

A noteworthy characteristic of our cohort is that a subset of patients—predominantly in the unsuccessful withdrawal group—had baseline TSH levels exceeding 10 mIU/L at initial diagnosis (median baseline TSH in the unsuccessful group: 12.67 mIU/L [IQR 6.35–23.45]), reflecting varying degrees of thyroid dysfunction at the time of treatment initiation. Current evidence suggests that patients with initial TSH levels above 10 mIU/L represent a population with more advanced thyroid gland impairment and substantially lower probability of spontaneous functional normalization after L-T4 withdrawal (20). This observation raises an important contextual point: in clinical practice, not all patients in our cohort may have had a clearly indicated diagnosis requiring permanent replacement therapy at the outset. Indeed, growing evidence indicates that a considerable proportion of patients initiating L-T4 therapy in routine practice have near-normal or borderline TSH levels, potentially reflecting diagnostic uncertainty or inappropriate prescribing (12). Patients in this category, who may have experienced transient or mild thyroid dysfunction, are more likely to maintain euthyroidism after drug discontinuation—consistent with the lower baseline TSH observed in our successful withdrawal group. The inclusion of patients with higher initial TSH levels in the unsuccessful withdrawal group may therefore reflect both genuine irreversible thyroid failure and a subgroup whose therapy was possibly initiated without robust evidence of permanent dysfunction. This clinical heterogeneity within the cohort should be considered when interpreting the overall withdrawal success rate (35.5%) and when applying the prediction model in clinical settings. Future studies should prospectively stratify patients by baseline TSH at diagnosis (e.g., <5, 5–10, and >10 mIU/L) to delineate withdrawal candidates more precisely and to evaluate whether the predictive model performs differentially across these subgroups.

Other studies have suggested that shorter treatment duration and lower pre-withdrawal L-T4 dose may favor withdrawal success ^18]^. In our cohort study, neither treatment duration nor L-T4 dose was significantly associated with withdrawal outcome, potentially reflecting differences in age distribution, sample size, and follow-up across studies. These results also suggest that treatment intensity or duration alone may not be the core determinant of withdrawal success.

An additional limitation is that the median L-T4 maintenance dose in our cohort was approximately 50 μg/day—relatively low compared with typical maintenance doses in patients with established overt hypothyroidism. Since L-T4 dose is generally inversely related to residual thyroid reserve and is considered a potential predictor of withdrawal success (18), the null association observed in our study likely reflects truncated dose variation rather than a genuine absence of a dose–outcome relationship. The findings may not generalize to populations receiving higher maintenance doses, and the contribution of L-T4 dosage to withdrawal outcomes warrants evaluation in more dosage-heterogeneous cohorts.

### 4.6 Clinical implications and model stability

We developed a clinically applicable model incorporating age, hypothyroidism classification, TPOAb status, and thyroid ultrasound echotexture to evaluate the probability of successful L-T4 withdrawal in primary hypothyroidism. The model showed excellent discrimination (AUC = 0.899), good calibration (Hosmer–Lemeshow P = 0.709), and strong internal stability (bootstrap MAE = 0.029). The nomogram format facilitates bedside use and supports individualized risk stratification when considering a supervised withdrawal trial.

From a clinical perspective, decisions regarding L-T4 withdrawal are often experience-driven and lack objective quantitative support. By integrating readily available clinical, serologic, and imaging variables, our model provides an interpretable estimate of withdrawal success probability, which may help identify candidates for withdrawal trials, reduce unnecessary long-term therapy, and optimize follow-up intensity and healthcare resource allocation.

### 4.7 Innovations and limitations

This study has several strengths. First, we integrated routine clinical features with thyroid ultrasound structural assessment to develop an interpretable prediction model and translated it into a nomogram for practical use. Second, internal validation using bootstrap resampling demonstrated good calibration and stability, supporting the reliability of model performance within the study cohort. Third, beyond immunologic indicators, we confirmed the independent prognostic value of ultrasound-defined structural abnormalities, adding an imaging dimension to withdrawal assessment.

The study has several limitations. First, as a single-center retrospective cohort study, it is inherently susceptible to selection and information bias; the modest sample size may further limit the precision of effect estimates and the generalizability of findings. Second, external validation was not performed, and the model’s transportability to other clinical settings and patient populations remains to be established. Third, follow-up was limited to 12 months, precluding assessment of longer-term recurrence risk after withdrawal. Fourth, the median treatment duration at the time of withdrawal was approximately 22–26 months, substantially shorter than the decades-long therapy experienced by many patients with established primary hypothyroidism in real-world practice. Whether the predictors and model performance identified here apply to patients with prolonged treatment histories remains uncertain, and this should be considered when extrapolating findings to longer-treated populations. Fifth, patients with severe comorbidities, malignancy, or concurrent use of thyroid-interfering medications were excluded per the study’s eligibility criteria. While these exclusions enhance internal validity by reducing confounding, the resulting cohort may not represent the broader clinical spectrum of patients with primary hypothyroidism. Clinicians should exercise caution when applying the model to patients outside these eligibility criteria. Sixth, patients excluded under criterion 7 (missing key laboratory or follow-up data; n=9) represent a potential source of systematic internal bias. Patients lost to follow-up may disproportionately include those who experienced early relapse and discontinued clinic attendance, or conversely, those who maintained stable euthyroidism and self-discontinued monitoring—either scenario would affect the estimated withdrawal success rate in opposite directions. The directionality of this attrition bias cannot be determined from the available data. Future studies should implement proactive follow-up protocols, including standardized telephone follow-up and pharmacy record linkage, to minimize loss to follow-up and enable sensitivity analyses comparing baseline characteristics between retained and excluded patients. Seventh, the uniform institutional TSH upper reference limit (4.78 mIU/L) applied in this study does not account for the well-established physiological rise in serum TSH with advancing age reference number for (29). In older adults, particularly those aged ≥65 years, a mildly elevated post-withdrawal TSH may fall within age-specific normative ranges and may not represent clinically meaningful relapse. Consequently, a subset of patients in the unsuccessful withdrawal group—especially those of advanced age—may have been misclassified under the uniform reference criteria used here. A sensitivity analysis applying age-adjusted TSH thresholds would be methodologically informative but could not be performed in the present study for three reasons: (1) the subgroup of patients aged ≥65 years was insufficiently large for a statistically robust analysis; (2) no locally validated age-stratified TSH reference ranges have been established for the institutional assay platform used; and (3) the retrospective design precluded prospective application of age-specific classification criteria. Future prospective studies enrolling larger age-stratified samples and employing assay-specific age-adjusted reference intervals are needed to determine whether withdrawal success rates differ meaningfully when age-appropriate TSH thresholds are applied. Further studies should include multi-center prospective cohort studies to externally validate and refine the model, incorporate additional continuous predictors (e.g., TSH trajectories, antibody titers, and thyroid volume), and explore whether non-linear machine learning approaches improve performance in heterogeneous populations.

A practical consideration for model implementation is that thyroid ultrasound and TPOAb measurement are not universally performed at the time of L-T4 initiation, particularly in resource-limited settings or when hypothyroidism is initially managed empirically. Application of the proposed nomogram would therefore require obtaining both tests prospectively prior to any withdrawal trial, which may increase upfront diagnostic costs. This should be factored into cost-effectiveness assessments before broader clinical adoption.

## 5 Conclusion

This study identified hypothyroidism classification (subclinical), age, TPOAb status, and thyroid ultrasound echotexture as independent factors associated with successful L-T4 withdrawal in primary hypothyroidism. The nomogram derived from these variables demonstrated good discrimination (AUC = 0.899) and calibration, and may facilitate pre-withdrawal risk stratification, follow-up planning, and individualized decision support. External validation in multi-center cohorts is warranted to confirm generalizability and to support broader clinical implementation.

## Data Availability

The original contributions presented in the study are included in the article/**Supplementary Material**. Further inquiries can be directed to the corresponding author.

## References

[1] McDermott MT . Hypothyroidism. Ann Intern Med. (2020) 173:ITC1–ITC16. doi: 10.7326/aitc202007070 32628881

[2] Zamwar UM Muneshwar KN . Epidemiology, types, causes, clinical presentation, diagnosis, and treatment of hypothyroidism. Cureus. (2023) 15(9):e46241. doi: 10.7759/cureus.46241 37908940 PMC10613832

[3] Jonklaas J Bianco AC Bauer AJ Burman KD Cappola AR Celi FS . Guidelines for the treatment of hypothyroidism: prepared by the American Thyroid Association task force on thyroid hormone replacement. Thyroid. (2014) 24:1670–751. doi: 10.1089/thy.2014.0028 25266247 PMC4267409

[4] Calissendorff J Falhammar H . To treat or not to treat subclinical hypothyroidism, what is the evidence? Medicina. (2020) 56:40. doi: 10.3390/medicina56010040 31963883 PMC7022757

[5] Ross DS . Treating hypothyroidism is not always easy: When to treat subclinical hypothyroidism, TSH goals in the elderly, and alternatives to levothyroxine monotherapy. J Internal Med. (2022) 291:128–40. doi: 10.1111/joim.13410 34766382

[6] Hepp Z Wyne K Manthena SR Wang S Gossain V . Adherence to thyroid hormone replacement therapy: a retrospective, claims database analysis. Curr Med Res Opin. (2018) 34:1673–8. doi: 10.1080/03007995.2018.1486293 29874941

[7] Ghamri R Babaker R Ezzat S Alsaedi H Alkhamisi M Arbaein R . Assessment of quality of life among patients with primary hypothyroidism: a case-control study. Cureus. (2022) 14(10):e29947. doi: 10.7759/cureus.29947 36348860 PMC9635404

[8] Ravensberg AJ Poortvliet RKE Du Puy RS Dekkers OM Mooijaart SP Gussekloo J . Effects of discontinuation of levothyroxine treatment in older adults: protocol for a self-controlled trial. BMJ Open. (2023) 13:e070741. doi: 10.1136/bmjopen-2022-070741 37185193 PMC10151847

[9] Rodriguez-Gutierrez R Maraka S Ospina NS Montori VM Brito JP . Levothyroxine overuse: time for an about face? Lancet Diabetes Endocrinol. (2017) 5:246–8. doi: 10.1016/s2213-8587(16)30276-5 28029536

[10] Lee SY Pearce EN . Hyperthyroidism: a review. JAMA. (2023) 330:1472–83. doi: 10.1001/jama.2023.19052 PMC10873132 37847271

[11] Fang H Zhao R Cui S Wan WQ . Sex differences in major cardiovascular outcomes and fractures in patients with subclinical thyroid dysfunction: a systematic review and meta-analysis. Aging (Albany NY). (2022) 14:8448. doi: 10.18632/aging.204352 36287183 PMC9648794

[12] Brito JP Ross JS El Kawkgi OM Maraka S Deng Y Shah ND . Levothyroxine use in the United States, 2008-2018. JAMA Intern Med. (2021) 181:1402–5. doi: 10.1001/jamainternmed.2021.2686 34152370 PMC8218227

[13] Bekkering GE Agoritsas T Lytvyn L Heen AF Feller M Moutzouri E . Thyroid hormones treatment for subclinical hypothyroidism: a clinical practice guideline. BMJ. (2019) 365:l2006. doi: 10.1136/bmj.l2006 31088853

[14] Maraka S Owen RR Singh Ospina NM Knox M Dodds T Thostenson JD . Discontinuation of levothyroxine therapy in patients with subclinical hypothyroidism: a pilot randomized clinical trial. Endocrine. (2025) 90(2):781–92. doi: 10.1007/s12020-025-04371-z 40736623 PMC12364413

[15] Livadas S Bothou C Androulakis I Boniakos A Angelopoulos N Duntas L . Levothyroxine replacement therapy and overuse: a timely diagnostic approach. Thyroid. (2018) 28:1580–6. doi: 10.1089/thy.2018.0014 30351232

[16] Rosario PW Calsolari MR . Levothyroxine therapy in the subclinical hypothyroidism: a lifelong therapy? A long‐term study. Clin Endocrinol. (2016) 85:819–20. doi: 10.1111/j.1365-2265.2009.03711.x 27515774

[17] Livadas S Angelopoulos N Kollias A Paparodis RD Androulakis I Anagnostis P . Thyroxine overuse and clinical indices guiding successful treatment withdrawal. J Endocrinol Invest. (2025) 48:1139–47. doi: 10.1007/s40618-025-02543-2 39899246

[18] Jung KY Kim H Choi HS An JH Cho SW Kim HJ . Clinical factors predicting the successful discontinuation of hormone replacement therapy in patients diagnosed with primary hypothyroidism. PLoS One. (2020) 15:e0233596. doi: 10.1371/journal.pone.0233596 32469958 PMC7259697

[19] Radetti G Salerno M Guzzetti C Cappa M Corrias A Cassio A . Thyroid function in children and adolescents with Hashimoto's thyroiditis after l-thyroxine discontinuation. Endocrine Connections. (2017) 6:206–12. doi: 10.1530/ec-17-0023 28348002 PMC5434746

[20] Burgos N Toloza FJK Singh Ospina NM Brito JP Salloum RG Hassett LC . Clinical outcomes after discontinuation of thyroid hormone replacement: a systematic review and meta-analysis. Thyroid. (2021) 31:740–51. doi: 10.1089/thy.2020.0679 33161885 PMC8110016

[21] Kim MJ Lee YJ Choe Y Shin CH Lee YA . Predictors for thyroid dysfunction after discontinuation of levothyroxine in children and adolescents with Hashimoto thyroiditis. Ann Pediatr Endocrinol Metab. (2024) 29:337–43. doi: 10.6065/apem.2346204.102 39506347 PMC11541090

[22] Radetti G Gottardi E Bona G Corrias A Salardi S Loche S . The natural history of euthyroid Hashimoto's thyroiditis in children. J Pediatr. (2006) 149:827–32. doi: 10.1016/j.jpeds.2006.08.045 17137901

[23] Radetti G Maselli M Buzi F Corrias A Mussa A Cambiaso P . The natural history of the normal/mild elevated TSH serum levels in children and adolescents with Hashimoto’s thyroiditis and isolated hyperthyrotropinaemia: a 3-year follow-up. Clin Endocrinol. (2012) 76:394–8. doi: 10.1111/j.1365-2265.2011.04251.x 21981142

[24] Crisafulli G Aversa T Zirilli G Pajno GB Corica D De Luca F . Subclinical hypothyroidism in children: when a replacement hormonal treatment might be advisable. Front Endocrinol. (2019) 10:109. doi: 10.3389/fendo.2019.00109 30858827 PMC6397829

[25] van der Spoel E van Vliet NA Poortvliet RKE Du Puy RS den Elzen WPJ Quinn TJ . Incidence and determinants of spontaneous normalization of subclinical hypothyroidism in older adults. J Clin Endocrinol Metab. (2024) 109:e1167–74. doi: 10.1210/clinem/dgad623 37862463 PMC10876405

[26] Livadas S Androulakis I Kollias A Paparodis RD Angelopoulos N Boniakos A . LBSAT247 Factors guiding successful treatment discontinuation in levothyroxine replacement therapy overuse: short and long-term observation data of 802 subjects. J Endocr Soc. (2022) 6:A745. doi: 10.1210/jendso/bvac150.1537

[27] Mammen JS . Understanding next steps to get the medical community going with deprescribing thyroid hormone. Clin Thyroidology®. (2025) 37:45–8. doi: 10.1089/ct.2025;37.45-48 36698420

[28] Chinese Society of Endocrinology . Guidelines for the diagnosis and treatment of adult hypothyroidism. Chin J Endocrinol Metab. (2017) 33:167–80. doi: 10.3760/cma.j.issn.1000-6699.2017.02.018 30704229

[29] Jansen HI Dirks NF Hillebrand JJ Ten Boekel E Brinkman JW Buijs MM . Age-specific reference intervals for thyroid-stimulating hormones and free thyroxine to optimize diagnosis of thyroid disease. Thyroid. (2024) 34:1346–55. doi: 10.1089/thy.2024.0346 39283820

[30] Shin DY Kim EK Lee EJ . Role of ultrasonography in outcome prediction in subclinical hypothyroid patients treated with levothyroxine. Endocrine J. (2010) 57:15–22. doi: 10.1507/endocrj.k09e-154 19823000

