# Supplementary material for: Factors associated with levothyroxine withdrawal and development of a prediction model in primary hypothyroidism
Source: Front Endocrinol (Lausanne). 2026 Jul 21;17:1852620. doi: 10.3389/fendo.2026.1852620 (PMC13433232; doi:10.3389/fendo.2026.1852620)
Supplement: Supplementary file 1 [file Table1.docx]

**Supplementary Table 1. Variable coding scheme**

| Variable | Coding |
| --- | --- |
| Successful withdrawal | No=0, Yes=1 |
| Hypothyroidism Classification | Overt=0 (reference),  Subclinical=1 |
| Age (years) | Continuous |
| TSH | Continuous |
| TgAb positivity | Negative=0, Positive=1 |
| TPOAb positivity | Negative=0, Positive=1 |
| Heterogeneous echotexture | No=0, Yes=1 |
| Hypoechogenicity | No=0, Yes=1 |
