# Supplementary material for: Factors associated with levothyroxine withdrawal and development of a prediction model in primary hypothyroidism
Source: Front Endocrinol (Lausanne). 2026 Jul 21;17:1852620. doi: 10.3389/fendo.2026.1852620 (PMC13433232; doi:10.3389/fendo.2026.1852620)
Supplement: Supplementary file 2 [file Table2.docx]

**Supplementary Table 2. Predictors included in the final model and corresponding multivariable logistic regression results**

| Predictor | B | SE | Wald χ² | P value | OR (95%CI) |
| --- | --- | --- | --- | --- | --- |
| Age (per year) | -0.103 | 0.027 | 14.393 | ＜0.001 | 0.903(0.856,0.952) |
| Classification  TPOAb | 1.853  -1.748 | 0.589  0.606 | 9.892  8.314 | 0.002  0.004 | 6.377(2.010,20.232)  0.174(0.053,0.571) |
| Heterogeneous echotexture | -2.213 | 0.663 | 11.137 | ＜0.001 | 0.109(0.030,0.410) |
| Constant | 7.449 | 1.914 | 15.151 | ＜0.001 |  |
