# Supplementary material for: Factors associated with levothyroxine withdrawal and development of a prediction model in primary hypothyroidism
Source: Front Endocrinol (Lausanne). 2026 Jul 21;17:1852620. doi: 10.3389/fendo.2026.1852620 (PMC13433232; doi:10.3389/fendo.2026.1852620)
Supplement: Supplementary file 3 [file Table3.docx]

**Supplementary Table 3. Multivariable Logistic Regression Analysis for Successful L-T4 withdrawal**

| Factor | *B* | *SE* | *Wald χ^2^* | *P* | *OR(95% CI)* |
| --- | --- | --- | --- | --- | --- |
| Hypothyroidism Classification |  |  |  |  |  |
| Overt Hypothyroidism | - | - | - | - | *ref* |
| Subclinical Hypothyroidism | 1.585 | 0.658 | 5.800 | 0.016 | 4.879(1.343–17.723) |
| Age | -0.104 | 0.028 | 13.405 | < 0.001 | 0.901(0.852–0.953) |
| TSH | -0.015 | 0.023 | 0.420 | 0.517 | 0.985(0.943–1.03) |
| TgAb |  |  |  |  |  |
| Negative | - | - | - | - | *ref* |
| Positive | -0.016 | 0.765 | 0.000 | 0.983 | 0.984(0.22–4.405) |
| TPOAb |  |  |  |  |  |
| Negative | - | - | - | - | *ref* |
| Positive | -1.897 | 0.668 | 8.063 | 0.005 | 0.150(0.040–0.556) |
| Heterogeneous echotexture |  |  |  |  |  |
| No | - | - | - | - | *ref* |
| Yes | -1.863 | 0.805 | 5.361 | 0.021 | 0.155(0.032–0.751) |
| Hypoechogenicity |  |  |  |  |  |
| No | - | - | - | - | *ref* |
| Yes | -0.791 | 0.627 | 1.589 | 0.207 | 0.453(0.133–1.551) |
| Constant | 6.570 | 2.268 | 8.392 | 0.004 | - |
